# Bis[2-(cyclo­propyl­imino­meth­yl)-5-methoxy­phenolato]zinc(II)

**DOI:** 10.1107/S1600536810013541

**Published:** 2010-04-17

**Authors:** Li-Ming Bai

**Affiliations:** a College of Chemistry and Chemical Engineering, Qiqihar University, Qiqihar 161006, People’s Republic of China

## Abstract

In the title complex, [Zn(C_11_H_12_NO_2_)_2_], the Zn^2+^ ion (site symmetry 2) is coordinated by two *N*,*O*-bidentate Schiff base ligands, generating a tetra­hedral ZnO_2_N_2_ geometry for the metal ion.

## Related literature

For background to zinc complexes with Schiff bases, see: Maxim *et al.* (2008 ▶); Ali *et al.* (2004 ▶); Keypour *et al.* (2009 ▶); Osowole *et al.* (2008 ▶); Kulandaisamy & Thomas (2008 ▶). For related structures, see: Wei *et al.* (2007 ▶); Li & Zhang (2005 ▶); Parvez & Birdsall (1990 ▶); Cui *et al.* (2009 ▶).
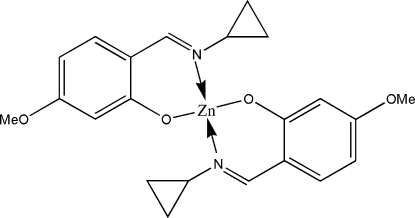


## Experimental

### 

#### Crystal data

[Zn(C_11_H_12_NO_2_)_2_]
*M* *_r_* = 445.80
Orthorhombic, 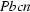

*a* = 8.9646 (18) Å
*b* = 10.628 (2) Å
*c* = 22.366 (4) Å
*V* = 2130.9 (7) Å^3^
*Z* = 4
Mo *K*α radiation
μ = 1.18 mm^−1^
*T* = 298 K
0.23 × 0.21 × 0.20 mm

#### Data collection

Bruker SMART CCD diffractometer
Absorption correction: multi-scan (*SADABS*; Sheldrick, 1996 ▶) *T*_min_ = 0.773, *T*_max_ = 0.798
12146 measured reflections
2424 independent reflections
1492 reflections with *I* > 2σ(*I*)
*R*_int_ = 0.050

#### Refinement

*R*[*F* ^2^ > 2σ(*F* ^2^)] = 0.042
*wR*(*F* ^2^) = 0.126
*S* = 1.02
2424 reflections
133 parameters
H-atom parameters constrained
Δρ_max_ = 0.43 e Å^−3^
Δρ_min_ = −0.31 e Å^−3^

### 

Data collection: *SMART* (Bruker, 1998 ▶); cell refinement: *SAINT* (Bruker, 1998 ▶); data reduction: *SAINT*; program(s) used to solve structure: *SHELXS97* (Sheldrick, 2008 ▶); program(s) used to refine structure: *SHELXL97* (Sheldrick, 2008 ▶); molecular graphics: *SHELXTL* (Sheldrick, 2008 ▶); software used to prepare material for publication: *SHELXTL*.

## Supplementary Material

Crystal structure: contains datablocks global, I. DOI: 10.1107/S1600536810013541/hb5404sup1.cif

Structure factors: contains datablocks I. DOI: 10.1107/S1600536810013541/hb5404Isup2.hkl

Additional supplementary materials:  crystallographic information; 3D view; checkCIF report

## Figures and Tables

**Table:** 

Zn1—O1	1.9169 (19)
Zn1—N1	2.017 (3)

**Table:** 

O1—Zn1—O1^i^	117.19 (11)
O1—Zn1—N1^i^	117.02 (10)
O1—Zn1—N1	97.10 (9)

Symmetry code: (i) 
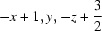
.

**Fig. 1.**
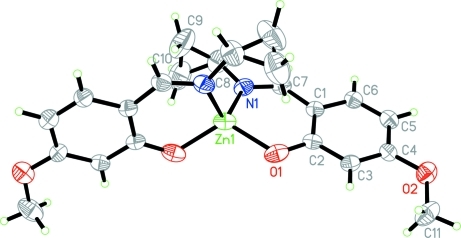
The molecular structure of (I), showing 30% probability displacement ellipsoids. Unlabeled atoms are at the symmetry position 1 - x, y, 3/2 - z.

## supplementary crystallographic information

### Comment

Zinc complexes with Schiff bases have attracted much attention in coordination chemistry and biological chemistry (Maxim *et al.*, 2008; Ali *et al.*, 2004; Keypour *et al.*, 2009; Osowole *et al.*, 2008; Kulandaisamy & Thomas, 2008). In the present paper, the title zinc(II) complex with the Schiff base 2-(cyclopropyliminomethyl)-5-methoxyphenol has been prepared and characterized by X-ray diffraction.

The title zinc complex, Fig. 1, possesses crystallographic two-fold rotation axis symmetry. The Zn atom is coordinated by two phenolic oxygen and two imino N atoms from two Schiff base ligands, generating a tetrahedral geometry. The bond lengths and angles (Table 1) around the Zn atom are typical and comparable to those in other Schiff base zinc(II) complexes (Wei *et al.*, 2007; Li & Zhang, 2005; Parvez & Birdsall, 1990; Cui *et al.*, 2009).

### Experimental

2-Hydroxy-4-methoxybenzaldehyde (0.152 g, 1 mmol) and cyclopropylamine (0.057 g, 1 mmol) were mixed and refluxed in a methanol solution (50 ml) with stirring for 1 h. To the above solution was added a methanol solution (10 ml) of Zn(CH_3_COO)_2_.2H_2_O (0.110 g, 0.5 mmol). The mixture was stirred at reflux for another 1 h, and cooled to room temperature. After keeping the solution in air for a few days, colourless blocks of (I) were formed.

### Refinement

Hydrogen atoms were placed in calculated positions and constrained to ride on their parent atoms with C–H distances in the range 0.93-0.97 Å, and with U_iso_(H) set at 1.2U_eq_(C) and 1.5U_eq_(methyl C).

### Crystal data

**Table:**

[Zn(C_11_H_12_NO_2_)_2_]	*F*(000) = 928
*M**_r_* = 445.80	*D*_x_ = 1.390 Mg m^−^^3^
Orthorhombic, *P**b**c**n*	Mo *K*α radiation, λ = 0.71073 Å
Hall symbol: -P 2n 2ab	Cell parameters from 1989 reflections
*a* = 8.9646 (18) Å	θ = 2.7–24.5°
*b* = 10.628 (2) Å	µ = 1.18 mm^−^^1^
*c* = 22.366 (4) Å	*T* = 298 K
*V* = 2130.9 (7) Å^3^	Block, colourless
*Z* = 4	0.23 × 0.21 × 0.20 mm

### Data collection

**Table:**

Bruker SMART CCD diffractometer	2424 independent reflections
Radiation source: fine-focus sealed tube	1492 reflections with *I* > 2σ(*I*)
graphite	*R*_int_ = 0.050
ω scans	θ_max_ = 27.5°, θ_min_ = 1.8°
Absorption correction: multi-scan (*SADABS*; Sheldrick, 1996)	*h* = −7→11
*T*_min_ = 0.773, *T*_max_ = 0.798	*k* = −12→13
12146 measured reflections	*l* = −28→28

### Refinement

**Table:**

Refinement on *F*^2^	Primary atom site location: structure-invariant direct methods
Least-squares matrix: full	Secondary atom site location: difference Fourier map
*R*[*F*^2^ > 2σ(*F*^2^)] = 0.042	Hydrogen site location: inferred from neighbouring sites
*wR*(*F*^2^) = 0.126	H-atom parameters constrained
*S* = 1.02	*w* = 1/[σ^2^(*F*_o_^2^) + (0.0608*P*)^2^ + 0.4149*P*] where *P* = (*F*_o_^2^ + 2*F*_c_^2^)/3
2424 reflections	(Δ/σ)_max_ < 0.001
133 parameters	Δρ_max_ = 0.43 e Å^−^^3^
0 restraints	Δρ_min_ = −0.31 e Å^−^^3^

### Special details

**Table:**

Geometry. All esds (except the esd in the dihedral angle between two l.s. planes) are estimated using the full covariance matrix. The cell esds are taken into account individually in the estimation of esds in distances, angles and torsion angles; correlations between esds in cell parameters are only used when they are defined by crystal symmetry. An approximate (isotropic) treatment of cell esds is used for estimating esds involving l.s. planes.
Refinement. Refinement of *F*^2^ against ALL reflections. The weighted *R*-factor wR and goodness of fit *S* are based on *F*^2^, conventional *R*-factors *R* are based on *F*, with *F* set to zero for negative *F*^2^. The threshold expression of *F*^2^ > σ(*F*^2^) is used only for calculating *R*-factors(gt) etc. and is not relevant to the choice of reflections for refinement. *R*-factors based on *F*^2^ are statistically about twice as large as those based on *F*, and *R*- factors based on ALL data will be even larger.

### Fractional atomic coordinates and isotropic or equivalent isotropic displacement parameters (Å^2^)

**Table:**

	*x*	*y*	*z*	*U*_iso_*/*U*_eq_	
Zn1	0.5000	0.55230 (4)	0.7500	0.0614 (2)	
N1	0.3162 (3)	0.4471 (2)	0.73578 (11)	0.0623 (7)	
O1	0.4246 (2)	0.64630 (17)	0.81662 (9)	0.0670 (6)	
O2	0.1210 (2)	0.7046 (2)	0.98928 (9)	0.0731 (6)	
C1	0.2075 (3)	0.5152 (2)	0.83071 (12)	0.0514 (7)	
C2	0.3079 (3)	0.6121 (2)	0.84814 (12)	0.0516 (7)	
C3	0.2785 (3)	0.6755 (2)	0.90190 (12)	0.0552 (7)	
H3	0.3418	0.7402	0.9139	0.066*	
C4	0.1583 (3)	0.6445 (3)	0.93745 (12)	0.0545 (7)	
C5	0.0621 (4)	0.5468 (3)	0.92121 (13)	0.0585 (7)	
H5	−0.0177	0.5243	0.9455	0.070*	
C6	0.0882 (3)	0.4853 (3)	0.86889 (13)	0.0569 (7)	
H6	0.0240	0.4206	0.8579	0.068*	
C7	0.2158 (4)	0.4431 (2)	0.77678 (15)	0.0597 (7)	
H7	0.1386	0.3860	0.7706	0.072*	
C8	0.2943 (4)	0.3637 (4)	0.68479 (16)	0.0864 (10)	
H8	0.2062	0.3092	0.6868	0.104*	
C9	0.4218 (5)	0.3109 (5)	0.6553 (2)	0.1302 (19)	
H9A	0.4138	0.2255	0.6403	0.156*	
H9B	0.5198	0.3341	0.6700	0.156*	
C10	0.3341 (6)	0.4067 (5)	0.6265 (2)	0.1273 (17)	
H10A	0.3771	0.4901	0.6230	0.153*	
H10B	0.2711	0.3815	0.5933	0.153*	
C11	0.1961 (5)	0.8193 (3)	1.00303 (15)	0.0968 (12)	
H11A	0.3005	0.8029	1.0085	0.145*	
H11B	0.1555	0.8543	1.0391	0.145*	
H11C	0.1828	0.8778	0.9708	0.145*	

### Atomic displacement parameters (Å^2^)

**Table:**

	*U*^11^	*U*^22^	*U*^33^	*U*^12^	*U*^13^	*U*^23^
Zn1	0.0615 (4)	0.0490 (3)	0.0737 (4)	0.000	0.0197 (2)	0.000
N1	0.0615 (17)	0.0554 (14)	0.0701 (17)	0.0017 (12)	0.0063 (12)	−0.0116 (12)
O1	0.0642 (13)	0.0551 (11)	0.0817 (14)	−0.0132 (10)	0.0280 (11)	−0.0105 (10)
O2	0.0773 (15)	0.0828 (15)	0.0593 (13)	−0.0106 (12)	0.0155 (10)	−0.0056 (11)
C1	0.0463 (16)	0.0465 (14)	0.0613 (17)	0.0000 (12)	0.0041 (13)	0.0014 (13)
C2	0.0491 (16)	0.0423 (14)	0.0636 (17)	0.0024 (12)	0.0075 (13)	0.0035 (13)
C3	0.0554 (17)	0.0466 (15)	0.0634 (17)	−0.0047 (13)	0.0062 (13)	−0.0015 (13)
C4	0.0557 (17)	0.0542 (16)	0.0537 (16)	0.0038 (13)	0.0006 (13)	0.0056 (13)
C5	0.0490 (16)	0.0639 (18)	0.0625 (18)	−0.0016 (14)	0.0079 (14)	0.0116 (15)
C6	0.0468 (17)	0.0561 (17)	0.0677 (19)	−0.0050 (13)	0.0014 (13)	0.0055 (14)
C7	0.0516 (18)	0.0504 (16)	0.0771 (19)	−0.0014 (14)	0.0008 (16)	−0.0037 (15)
C8	0.074 (2)	0.102 (3)	0.083 (2)	0.007 (2)	0.0097 (19)	−0.021 (2)
C9	0.078 (3)	0.156 (4)	0.157 (4)	0.030 (3)	−0.009 (3)	−0.095 (4)
C10	0.127 (4)	0.164 (5)	0.091 (3)	−0.015 (4)	0.004 (3)	−0.027 (3)
C11	0.113 (3)	0.097 (3)	0.080 (3)	−0.024 (2)	0.021 (2)	−0.031 (2)

### Geometric parameters (Å, °)

**Table:**

Zn1—O1	1.9169 (19)	C5—C6	1.360 (4)
Zn1—O1^i^	1.9169 (19)	C5—H5	0.9300
Zn1—N1^i^	2.017 (3)	C6—H6	0.9300
Zn1—N1	2.017 (3)	C7—H7	0.9300
N1—C7	1.286 (4)	C8—C10	1.427 (5)
N1—C8	1.458 (4)	C8—C9	1.434 (5)
O1—C2	1.313 (3)	C8—H8	0.9800
O2—C4	1.366 (3)	C9—C10	1.439 (7)
O2—C11	1.426 (4)	C9—H9A	0.9700
C1—C6	1.405 (4)	C9—H9B	0.9700
C1—C2	1.422 (4)	C10—H10A	0.9700
C1—C7	1.431 (4)	C10—H10B	0.9700
C2—C3	1.403 (4)	C11—H11A	0.9600
C3—C4	1.380 (4)	C11—H11B	0.9600
C3—H3	0.9300	C11—H11C	0.9600
C4—C5	1.398 (4)		
			
O1—Zn1—O1^i^	117.19 (11)	C1—C6—H6	118.5
O1—Zn1—N1^i^	117.02 (10)	N1—C7—C1	128.3 (3)
O1^i^—Zn1—N1^i^	97.10 (9)	N1—C7—H7	115.9
O1—Zn1—N1	97.10 (9)	C1—C7—H7	115.9
O1^i^—Zn1—N1	117.02 (10)	C10—C8—C9	60.4 (3)
N1^i^—Zn1—N1	112.64 (14)	C10—C8—N1	119.1 (4)
C7—N1—C8	116.3 (3)	C9—C8—N1	119.4 (3)
C7—N1—Zn1	118.6 (2)	C10—C8—H8	115.6
C8—N1—Zn1	124.8 (2)	C9—C8—H8	115.6
C2—O1—Zn1	123.65 (17)	N1—C8—H8	115.6
C4—O2—C11	117.9 (2)	C8—C9—C10	59.6 (3)
C6—C1—C2	118.6 (3)	C8—C9—H9A	117.8
C6—C1—C7	115.5 (3)	C10—C9—H9A	117.8
C2—C1—C7	125.9 (3)	C8—C9—H9B	117.8
O1—C2—C3	118.5 (2)	C10—C9—H9B	117.8
O1—C2—C1	123.9 (2)	H9A—C9—H9B	115.0
C3—C2—C1	117.7 (2)	C8—C10—C9	60.0 (3)
C4—C3—C2	121.7 (3)	C8—C10—H10A	117.8
C4—C3—H3	119.1	C9—C10—H10A	117.8
C2—C3—H3	119.1	C8—C10—H10B	117.8
O2—C4—C3	124.7 (3)	C9—C10—H10B	117.8
O2—C4—C5	114.7 (3)	H10A—C10—H10B	114.9
C3—C4—C5	120.7 (3)	O2—C11—H11A	109.5
C6—C5—C4	118.3 (3)	O2—C11—H11B	109.5
C6—C5—H5	120.8	H11A—C11—H11B	109.5
C4—C5—H5	120.8	O2—C11—H11C	109.5
C5—C6—C1	123.1 (3)	H11A—C11—H11C	109.5
C5—C6—H6	118.5	H11B—C11—H11C	109.5

Symmetry codes: (i) −*x*+1, *y*, −*z*+3/2.
